# Quantum Cascade Laser-Based Infrared Microscopy for Label-Free and Automated Cancer Classification in Tissue Sections

**DOI:** 10.1038/s41598-018-26098-w

**Published:** 2018-05-16

**Authors:** Claus Kuepper, Angela Kallenbach-Thieltges, Hendrik Juette, Andrea Tannapfel, Frederik Großerueschkamp, Klaus Gerwert

**Affiliations:** 1 Chair of Biophysics, Faculty of Biology and Biotechnology, Ruhr University Bochum, Bochum, Germany; 2 Institute of Pathology, Ruhr University Bochum, Bochum, Germany

## Abstract

A feasibility study using a quantum cascade laser-based infrared microscope for the rapid and label-free classification of colorectal cancer tissues is presented. Infrared imaging is a reliable, robust, automated, and operator-independent tissue classification method that has been used for differential classification of tissue thin sections identifying tumorous regions. However, long acquisition time by the so far used FT-IR-based microscopes hampered the clinical translation of this technique. Here, the used quantum cascade laser-based microscope provides now infrared images for precise tissue classification within few minutes. We analyzed 110 patients with UICC-Stage II and III colorectal cancer, showing 96% sensitivity and 100% specificity of this label-free method as compared to histopathology, the gold standard in routine clinical diagnostics. The main hurdle for the clinical translation of IR-Imaging is overcome now by the short acquisition time for high quality diagnostic images, which is in the same time range as frozen sections by pathologists.

## Introduction

For more than 20 years many scientists have focused on the clinical translation of Fourier transform infrared (FTIR) based microscopy for tissue diagnostics^1–4^, primarily on the automated, label-free classification of tumorous tissue^5^, including, but not limited to, colorectal^6^, lung^7^, prostate^8^, and bladder^9^ cancer or melanoma^10^ tissues. These studies demonstrated sensitivity and specificity of this IR imaging technique higher than 90%, as compared to histopathological as well as immunohistochemical (IHC) diagnostics by pathologists. We previously implemented a technique for the automated label-free classification of colorectal cancers with high accuracy, sensitivity, and specificity (96% accuracy, 94% sensitivity, and 100% specificity) and in addition the differential cancer diagnosis, including its grading for FTIR imaging^11,12^. Furthermore, we analyzed lung and thoracic tumors, showing that FTIR imaging can be used for the identification of different lung cancer types, especially subtypes of adenocarcinomas with 96% accuracy and diffuse pleural malignant mesothelioma subclasses (sarcomatoid and epithelioid) with 88% accuracy^13^. Recently, by integrating FTIR imaging and laser capture microdissection (LCM), annotated tissue was cut out precisely and analyzed by proteomics providing in addition to spatial resolution also molecular resolution^14^. Thereby the respective vibrational spectra are assigned to specific protein alterations in the diseased tissue as compared to healthy tissue. These results demonstrate that FTIR imaging of tissue thin sections delivers already in one index color image the full information as biomarkers of multi-panel IHC staining’s, which are used to differentiate subtypes of cancer. This integrated approach of IR imaging guided microdissection and proteome analysis can be used to identify new protein biomarkers^14^. However, the clinical translation of this powerful technique is hindered mainly by the several hours long measuring time and the complex and large instrumental FTIR setup with liquid nitrogen cooling.

These issues can be overcome by using the quantum cascade laser (QCL) as high power light source instead of the globar in FTIR set-ups. The Bhargava and Petrich groups conducted pioneering work in the field of chemical imaging with home-made QCL based microscopes^15–17^. The initial studies using the first-generation commercially available Spero IR microscope (Daylight Solutions, San Diego, CA, USA), reported already promising results^18–20^. However, coherence effects, low laser stability, and offset edges in mosaic datasets due to insufficient movement accuracy of the stage had to be overcome. Here, transients due to laser stability and offset edges were minimized in an improved QCL based IR microscope (Spero QT). In combination with the new developed classification model of colorectal cancer tissue thin sections this allows spatially resolved, label-free, automated, and observer-/operator-independent annotation now within few minutes, the same time range used for a frozen section of pathologists. Furthermore, the presented feasibility study shows that coherence effects can be overcome by a well-trained classifier.

Over 60,000 new colorectal cancer cases in Germany and over 14 million worldwide were diagnosed in 2016^21,22^. The initial phase in the majority of these cases is the formation of adenomas with intraepithelial neoplasia. Colonoscopy allows initial detection of suspicious tumor regions. During colonoscopy biopsies were taken, and then thin sections of the biopsies were stained. The histopathological cancer diagnosis on stained thin sections is performed by the pathologists and determines the gold standard in the clinics. Here, we obtained images that show significant shorter data acquisition time for high quality IR images. In the same time range as a frozen thin section diagnosis by a pathologist. This allows now the application of QCL based IR imaging in large clinical studies and also for biomarker research.

We studied 100 samples with UICC Stage II and III colorectal cancer tissue and 20 tumor-free tissue samples of 110 randomly chosen patients older than 18 years and developed a workflow that enables the tissue classification for diagnosis in about 30 min for large thin sections, while smaller regions of interest can be analyzed within few minutes both with a sensitivity of 96% and specificity of 100% as compared to histopathology.

## Results

### Data acquisition for QCL based IR microscopes

In QCL based IR microscopes coherence effects occur due to the physical characteristics of laser light sources as compared to the classical black body light source like the globar used in FTIR based microscopes. These coherence effects as observed in previous studies hampered the correct annotation in the IR images. Two types of coherence effects sources must be discussed those caused by instruments optics (e.g. fringes) and those caused by the measured sample itself^23^. Both effects hamper the data acquisition. The instrument based coherence effects occurred due to laser instabilities that lead to differences between the background and sample measurements, resulting in sinus-shaped patterns and fringes, on the 480 × 480 camera. In the used QCL based microscope these coherence effects are now minimized by higher laser stability as shown in comparison with the former performance (Fig. S1). Mostly because the stability of the lasers has been improved, resulting in minimal variance between the measurements and a factor 100 lower noise level. The sample based coherence effects are still present and must be addressed in pre-processing and classification. The presented feasibility study proofs that this can be adequately addressed when the instruments coherence effects are minimized. Furthermore, the measuring time for a spectral hypercube was largely decreased to 47 s for a 2 × 2 mm field of view (FOV) with 2 cm^−1^ resolution from 1800–948 cm^−1^, enabling rapid data acquisition.

### Classifier for QCL based IR imaging

**Figure 1:**
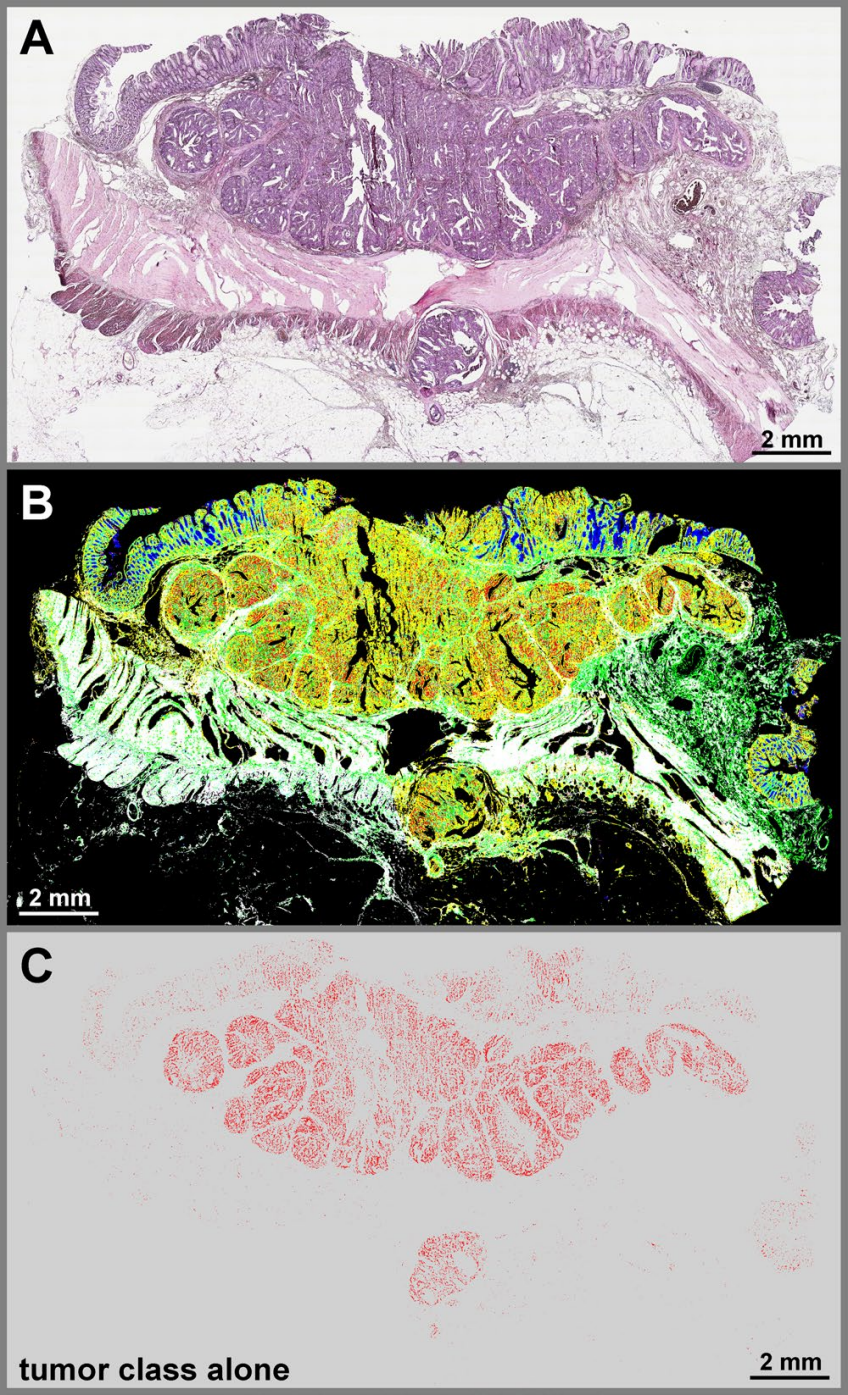
QCL based IR imaging of whole slice colorectal cancer tissue thin sections. Thin tissue section images of colorectal cancer marked by H&E in A and label-free index color images in B and C taken by QCL based IR microscope and analyzed by an improved new classifier. (**A**) H&E stained image of colorectal cancer thin section, showing morphological structures of diseased colon wall. Cancerous regions are prominently illustrated in the upper part by heavy purple hematoxylin staining patters. Below is the *muscularis propria* surrounded by connective tissue. Infiltrating inflammatory cells are distributed over the whole tissue sample. (**B**) index color image of the first RF classifier with the following tissue classes: red: pathological region; yellow: infiltrating inflammatory cells; white: muscle; green: connective tissue; cyan: crypts; blue: lumen. The morphological architecture is well represented by the index colors and agrees nicely with the annotation of two clinical pathologists. (**C**) index color image showing the tumor class only identified in the second classifier. The red pixels represent the cancerous region of the sample matching the annotation by the clinical pathologists.

Here, we developed and verified an automated label-free tissue classification workflow for colorectal cancer, examining 110 patient larger thin sections on PET frame slides using two QCL based IR imaging systems. Based on the large data sets obtained in short time the bioinformatic analysis is now largely improved. In deviation to our previous FTIR studies using Kevley lowE slides the data set is taken on tissue thin sections deposited now on PET frame slides. Therefore, a new RF classifier has to be implemented. The resulting automated label-free tissue classification allows reliable and robust annotations of larger tissue sections in 34 min with 4 cm^−1^ spectral resolution (Fig. 1). The annotations in the IR images match nicely those based on H&E staining comparing whole tissue thin sections. Pixel based analysis was not performed due to the large sample size and alterations occurring in the staining procedure, e.g. shrinking or detach of tissue parts. Since not the pixel but the overall diagnosis of the thin sections is of clinical relevance the statistics are provided based on the whole tissue thin section, but not on individual pixels.

The new QCL RF classifier is composed of two classifiers. The first RF discriminated healthy tissue types from pathologic. The pathologic spectra are subsequently presented to the second RF which discriminates cancerous regions. Possible variances occurring in the PET substrate between background and measurement as long as possible coherence on the sample were represented in the QCL RF training data.

The remarkable advantage of the QCL IR imaging is the gain of speed. Acquiring the FTIR image required 5400 min, with both measurements of similar spectral quality (spectral resolution of 4 cm^−1^) and almost similar pixel size (Spero QT, 4.2 µm; Cary 620, 5.5 µm). In this case the QCL based IR analyses were ~160 times faster than the FTIR based measurements for the same measured area. Considering the smaller pixel size of the Spero QT the speed-up is ~260 times. This gain of speed allows us to analyze a much larger number of patients in a much shorter time period. With FTIR imaging it would take over one year for the presented study whereas with QCL imaging all spectral datasets are taken in about 100 h with 2 cm^−1^ spectral resolution. Obtaining larger spectral data sets gives rise to much more accurate imaging classifiers because a much larger data set can be used for training and validation which represents the tumor heterogeneity and spectral distortions, e.g. coherence, much better.

Additionally, the IR images are compared to an adjacent tissue section on a Kevley IR reflective slide from the same patient using a state-of-the-art benchtop FTIR imaging system (Cary 620, Agilent Technologies, Santa Clara, CA, US), that was already used for tissue classification. Because the thin sections are deposited on different substrates the QCL and the FTIR RF classifier were not interchangeable. The interchangeability between the observed spectral classes and classifiers was previously discussed but cannot be addressed here due to different study design^23–25^. However, the comparison of the QCL and FTIR based images demonstrates convincingly, that the QCL IR imaging results are in nice agreement with those obtained using the FTIR imaging (Fig. S2). The observed deviations seem to be caused mainly by the use of an adjacent slice and the training of the FTIR classifier on samples of another study which could slightly differ in sample handling and processing. Furthermore, the previously FTIR classifier is performing the classification in one step which is less accurate by means of tumor detection. To compare the spectral quality, we measured the same sample with FTIR and QCL following the work of Wrobel *et al*.^24^. For almost the same tissue positions only slight differences occurred (Fig. S3). These could be caused by errors from image registration, the difference in pixel size, or possible coherence on the sample. Comparing spectral training classes of distinct tissue types underlines the comparability of the measured spectra for FTIR and QCL imaging (Fig. S8).

To illustrate the spectral quality of the QCL system the mean class spectra of pathological and connective tissue sections were obtained using the Spero QT (Fig. S4), and they were selected because colorectal cancer primarily develops from the epithelial cells. The standard deviation for the classes demonstrates the major variances in the training sets were obtained between 1300–1250 cm^−1^. This could be correlated to coherence effects on the sample as shown by Yeh *et al*. and must be addressed in the training data as shown here^23^. In addition, the visualization of the spectra illustrates that the transitions between the four laser modules do not affect the obtained spectral data (Fig. S5).

### Classification results of colorectal cancer

All 120 clinical tissue samples obtained from 110 randomly chosen patients were analyzed in less than 100 h using one Spero QT system. These results were validated using a second Spero QT instrument. Each slide was loaded with one sample. No tissue microarrays (TMA) were used as mostly in former studies, and all analyzed tissues were clinical routine samples. Using the approach, we developed, the sensitivity of 96% and the specificity of 100% were achieved based on the results obtained by analyzing 99 patient samples (true positives (TP): 78; false negatives (FN): 3; true negatives (TN): 18; false positives (FP): 0). The sum of red pixels, denoting tumor cells, must be above a chosen 2% threshold, since these signals may be obtained also due to spectral noise. Therefore, three false negatives were obtained. The three false negatives show in the H&E stained sections very low tumor content. The false negative tissue sections classified by IR imaging are from a later cut than the H&E stained sections and might therefore be located closer to the tumor border and might contain even less tumor cells than the H&E stained thin sections. With less tumor cells they fall below the 2% threshold. Using the same thin sections for IR imaging and H&E stained imaging would result in improved sensitivity and specificity, which is underestimated due study design. However, we do have to improve the quality of the data to lower further the threshold to come closer to 100%. This will be obtained in larger studies in the next step. Furthermore, in future studies the measurements of tissue from healthy colon, obtained from healthy patients, should be considered to proof the threshold. However, the label-free and automated workflow minimizes the inter- and intra-operator/observer variance. Samples of 18 patients are used for training data and are therefore not part of the statistical analysis. The constructed random forest (RF) classifier is schematically presented in Fig. S6.

**Figure 2:**
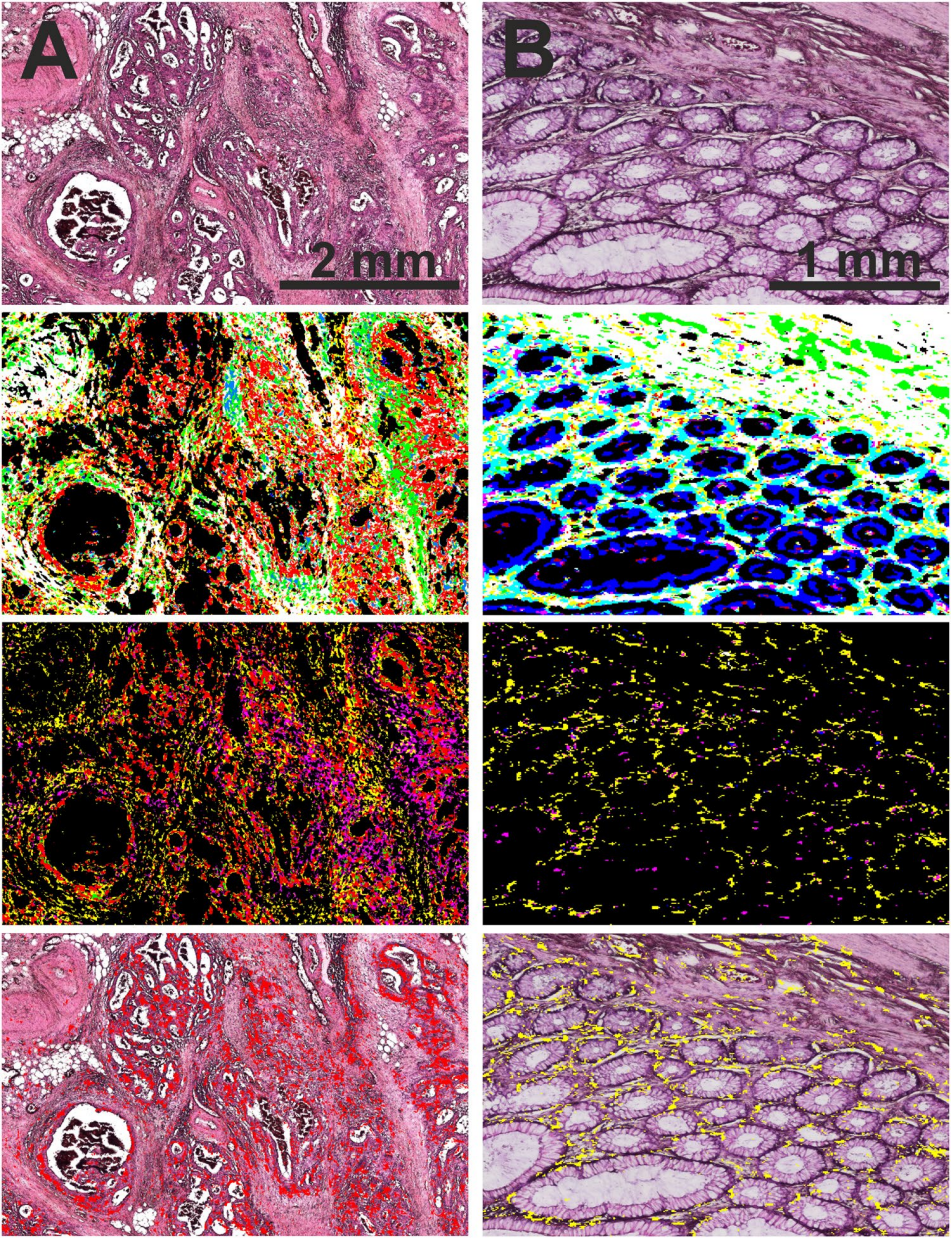
Detailed tissue classification with IR imaging in 100 seconds. Colorectal cancer tissue annotation provided by QCL based imaging: Cancerous (**A**) and tumor-free (**B**) tissue sections stained with H&E (top row), QCL based classification, showing the first RF (second row), the second RF (third row), and the overlay with the tumor class (**A**) and the infiltrating inflammatory cells (**B**) of the second RF analysis (fourth row). The QCL imaging tissue classification takes 100 seconds for each sample shown here. (**A**) The analysis of cancerous tissue sections, showing the tumor cells invading the connective tissue. second row: red, pathological regions; yellow, infiltrating inflammatory cells; white, *muscularis mucosae* and *muscularis propria*; green, connective tissue. Cell debris and blood are sorted out and presented in the background color black. All regions marked in red using the first level RF were further analyzed by the second level RF. third row: red, tumor cells; magenta, necrotic regions; yellow, infiltrating inflammatory cells. The inflammatory cells originate from the first level RF. The overlay of tumor class (red) and HE underlines the high spatial accuracy of detection (bottom row). (**B**) The analysis of tumor-free tissue sections. second row: cyan, healthy crypts; blue, lumen; white, thin muscular layer between the crypts and the submucosal connective tissue; green, connective tissue. The representative pathological, second level RF analysis is presented in the third row image: yellow, infiltrating inflammatory cells; magenta, necrosis; red, tumor cells. No tumor cells can be detected here. Following, the HE overlay is shown for the infiltrating inflammatory cells.

In Fig. 2, representative tissue regions are shown using the automatic label-free classification and the corresponding H&E-stained regions for comparison. A good agreement with the annotation provided by two clinical pathologists was achieved. Tumor cells were shown to invade the connective tissue (Fig. 2A), and the tissue architecture was clearly identifiable in these images. Additionally, inflammatory cells infiltrating the tumor site were observed as well. Because the diagnostics depends not on regions of cell debris and blood, these components are sorted out and assigned to the background color black. The pathological tissue regions were further classified using the second level RF, and the obtained images show a nice agreement with the results obtained using the H&E staining based established diagnosis. The annotation of infiltrating inflammatory cells shown in the second RF is obtained by the first RF. They remained unmodified by the second RF, but are shown in order to achieve a better morphological representation of the tissue.

In Fig. 2B, the analysis of a tumor-free tissue section is presented. In these images, the morphological layers of the colon wall can be observed, including the mucosa with healthy crypts and connective tissue, thin muscular layer (*lamina muscularis mucosae*), another layer of connective tissue (submucosa), and the muscular layer of the *muscularis propria*. The structures observed in these images were shown to match those observed using H&E stained sections. The morphological features were classified correctly as crypts, lumen, connective tissue, and musculature. In the second layer RF image (Fig. 2B third row), infiltrating inflammatory cells and few cells annotated as necrosis can also be observed. The presence of infiltrating inflammatory cells can be explained to the immune system activation in the colon, since these tumor-free samples were obtained from tumor patients. Colon tissue shows always inflammation so these classes were important to be properly represented. In the supplementary material we provided images of infiltrating inflammatory cells and lymph follicle in higher magnification to proof the correlation (Fig. S7). Furthermore, we show the variance of the spectral classes for this cell type in comparison to the FTIR spectra (Fig. S8). The spectra were quite similar which proofs the presence of such a high number of inflammatory cells.

In summary, the QCL based images shown in Fig. 2 provided a detailed automated tissue annotation in 100 seconds for regions of 2 × 4 mm. The resulting annotation is in agreement with the annotation by pathologists. No pixel based analysis was perfomed here since the large samples underly shrinkage and other effects in the staining procedure as mentioned before.

### Inter- and intra-observer/-operator variability

**Figure 3:**
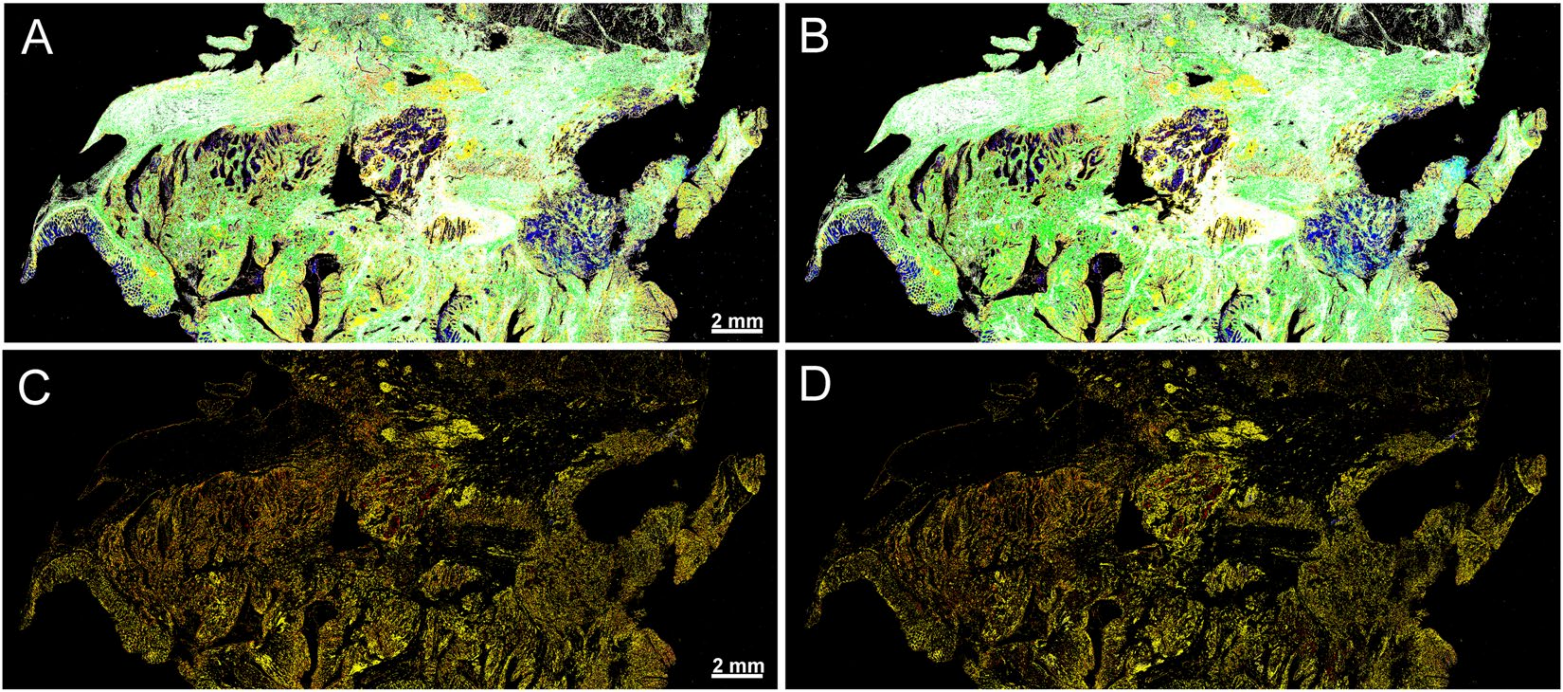
Transferability of the label-free tissue classification between different instruments. Two consecutive measurements of the same tissue section performed using two Spero QT microscopes. (**A**) Tissue section analysis using the first QCL based instrument. (**B**) Tissue section analysis using the second QCL based instrument: red, pathological regions; yellow, inflammatory cells; green, connective tissue; blue, lumen; cyan, crypts; white, musculature. (**C**) and (**D**) second RF for tumor detection with the first and the second QCL based instrument: red, tumor; yellow, infiltrating inflammatory cells; magenta, necrosis; green, inflammation. Only subtle are visible. Overall the detection matches very well.

We performed two consecutive analyses of the same sample using two Spero QT microscopes with randomized operators (Fig. 3), applying the same bioinformatic classifiers. Our results demonstrated that the obtained annotations were in full agreement, showing that inter- and intra-observer/-operator variability between different QCL based annotations is minimized. The subtle differences seen in the two images are due to the slightly decreased noise level obtained in the second microscope. The overall accordance for the shown sample is 94%. Main differences are occurring on the middle right tissue parts where the tissue was parted due to cutting. Furthermore, due to the laser module characteristics, an improved illumination of the detector was achieved in the second instrument, leading to even better quality of data. Nevertheless, pathological regions were identified the same using both microscopes. Taking into the consideration only the results obtained with one instrument, sensitivity of 96% and specificity of 100% were obtained for sample based annotation. Using the second instrument as an independent validation instrument, only sensitivity of 92% and the specificity of 100% were achieved for sample based annotation (TP: 35; FN: 3; TN: 18; FP: 0). The lower sample numbers resulted from later availability of the second instrument and some samples were no longer available. In this case the sensitivity is not a good measure because it depends on the sample number. In order to compare the performance of the two instruments the predictive value is a better measure. Both instruments yielded the same positive predictive value (PPV) with 100% and negative predictive value (NPV) with 86%. This shows that they have the same performance. Further improved specifications and workflow for the instruments will lead to even better comparability in future since the differences in illumination and user dependent differences in handling, e.g. focusing of the sample, could be minimized.

### Reducing the data acquisition time for clinical translation

However, for cancer pre-screening or routine clinical analyses the measuring time might need further reduction. In order to minimize the acquisition time and computational workload we reduced the spectral resolution. The RF is trained on unsmoothed spectra and as the spectral bands are very broad (Fig. S5) and do not need 2 cm^−1^ spectral resolution, it is stepwise reduced from 2 cm^−1^ to 4 cm^−1^, 8 cm^−1^, and 16 cm^−1^ and the respective implications for the classifications are analyzed. Due to the characteristics of the QCL system, the measurement time is linearly-dependent on the wavenumber resolution. Therefore, the reduction of spectral resolution to 16 cm^−1^ is equivalent to eight-fold shorter measuring time, suggesting that for the thin section presented in Fig. 1, only 8 min are required at 16 cm^−1^ resolution. Comparing the label-free classification performed using different resolutions demonstrated that even the reduction to 16 cm^−1^ did not considerably alter the quality of the obtained results (Fig. S9). The loss in quality down to 8 cm^−1^ is comparable to the differences seen between the two instruments shown before. With the 16 cm^−1^ the differences become more prominent and are occurring for infiltrating inflammatory cells and tumor regions which were overrepresented. By decreasing resolution, the specificity is reduced. However, in order to use the reduced spectral resolution, the spectral pre-processing has to be improved, which is not yet addressed here. Furthermore, by reducing spectral resolution, the data volume is linearly reduced (16 cm^−1^ is equivalent to 8 times less data amount compared to 2 cm^−1^ spectral resolution) and computational load is considerably decreased as well, resulting in significant shorter computing time.

**Figure 4:**
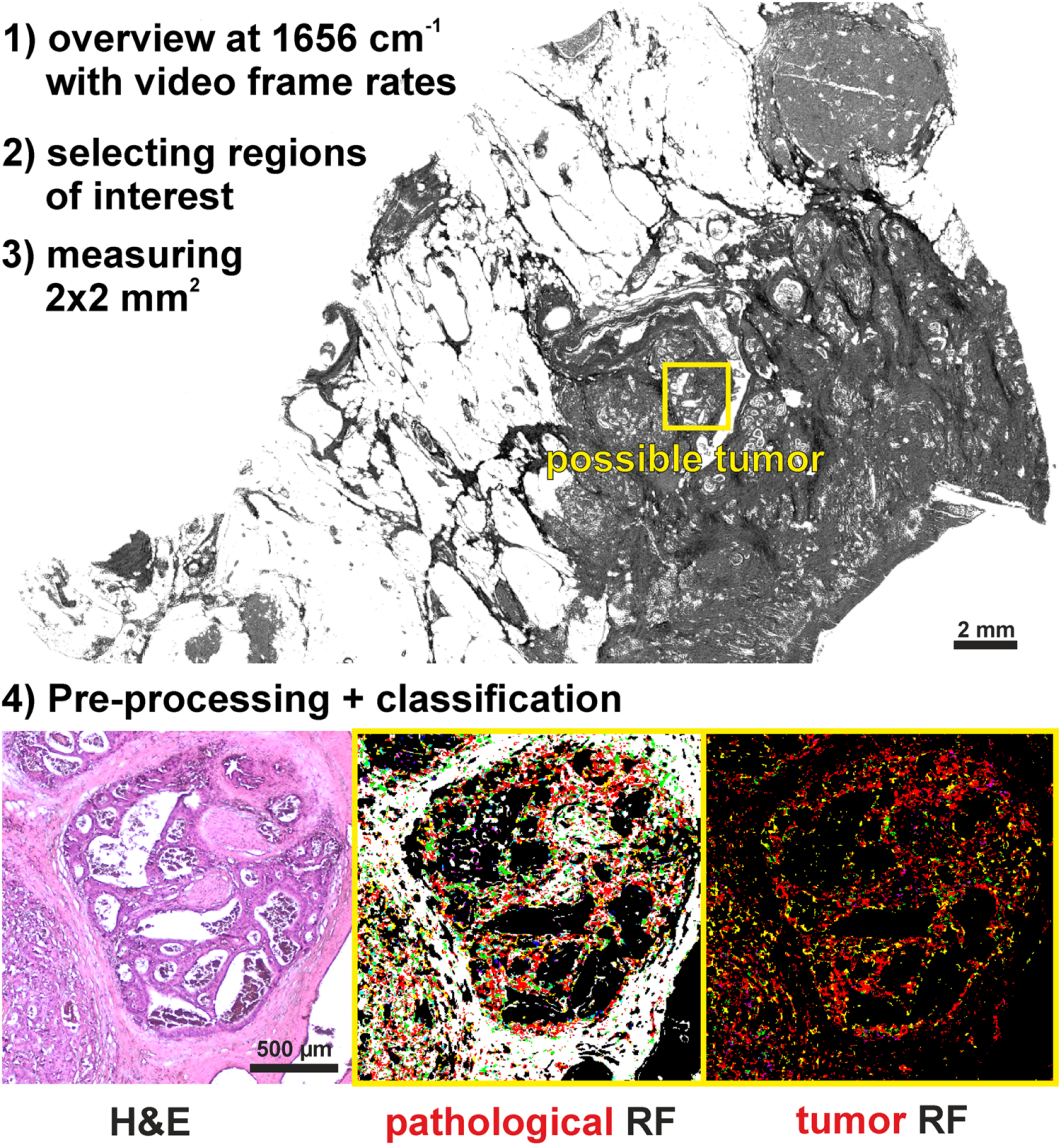
Suggested diagnostic workflow. The overview image was taken at amid I (1656 cm^−1^), showing tissue morphological characteristics. Upper right, a large lymph follicle; center, glandular tumor lesion; yellow rectangle, region of interest. Bottom, single field analyses. Left, H&E staining; middle, first-level RF analysis; red, pathological regions; yellow, the invading inflammatory cells; white, the surrounding muscles; green, connective tissue. Right, second level RF analysis. All regions marked red using the first level RF were further analyzed by the second level RF of the selected, single-field, region: red, tumor cells; magenta, necrotic regions; yellow, infiltrating inflammatory cells.

Furthermore, the measuring time can also be reduced by using a single wavelength with video frame rates for an overview image for the morphological preselection of tumorous regions, allowing thereby the identification of much smaller regions of interest in very short times. In Fig. 4, we present such an exemplary workflow. The overview of a sample with size 1.4 × 2 cm can be obtained using the amide I band at 1656 cm^−1^ within 1 min. Such overview is suitable for a trained pathologist to recognize suspicious areas. Afterward, a suspicious region of 2 × 2 mm can be classified in 50 s by measuring the entire spectral range with 2 cm^−1^ spectral resolution. In such approach the entire workflow from sample preparation to spectral diagnosis, can be performed within few minutes.

## Discussion

The major challenges for the translation of IR imaging into clinical use are the measuring time, usability, reliability, sensitivity and specificity, and instrumental complexity. However, sufficient reliability, sensitivity, and specificity have been demonstrated by our and other groups using FTIR based microscopes^6,11^. However, the measuring time and the usability of instruments in a clinical environment remained the most important issues. Previous QCL based studies^15–17^ showed that the high spectral brightness of the lasers leads to remarkable lower measuring times compared with that of the FTIR based microscopes. Furthermore, the QCLs allow the use of uncooled microbolometers as detectors and a much smaller instrument footprint. However, the QCL based microscopes used so far show large coherence effects occuring from instruments optics. Overcoming these issues is challenging, and therefore, the majority of the published QCL based IR imaging studies show the spectral data only for narrow wavenumber regions or distinct wavenumbers. First pioneering studies were done by the Petrich and the Bhargava group with inhouse developed QCL IR imaging systems. Recent developments in the field of QCL based IR imaging led to the release of a commercially available instrument, Spero, produced by Daylight Solutions, CA, USA, which can perform measurements from 1800 to 920 cm^−1^ with 4 cm^−1^ spacing. Several research groups, e.g. the Gardner goup, used this instrument in tissue analyses^18,26^, including a proof of principle analysis of two colorectal cancer tissue sections by Bird *et al*.^27^. This recent publications using the first generation Spero microscope demonstrated the applicability of QCL based IR microscopy to TMAs and fluid samples^27,28^. We considerably extended here the approach, not only by analyzing more patient samples, but we also annotate more different tissue types and morphological structures by a largely improved bioinformatics analysis providing a significantly improved classifier. The sample based diagnosis is in fully agreement with the diagnosis provided by two clinical pathologists. The tissue classification can be performed without inter- and intra-observer/operator variability. This has been validated using a second Spero QT QCL based IR imaging systems with sensitivity of 92% and specificity of 100% obtained in a second independent measurement set.

The measuring time with the QCL based microscope is 160 times faster as compared to FTIR-based microscopes with 4 cm^−1^ spectral resolution for the same measured area. Further acceleration can easily be obtained by using lower spectral resolution; 16 cm^−1^ instead of 4 cm^−1^ would reduce the measuring time by a factor 4. The presented results demonstrate an equally well performance using lower wavenumber resolution. In the previous studies by the Gardner group, still a significant decrease in accuracy for independent test sets due to lower spectral resolution was reported^19^. However, our approach is based on a much larger data set allowing now to perform a more detailed bioinformatic analysis, and to validate our results using an independent test set. Thus, the presented results imply that the reduction of spectral resolution will lead to shorter measuring times without loss of sensitivity and specificity for colorectal cancer by further improvement in the spectral pre-processing.

In addition, QCL based IR-microscopes allow in contrast to FTIR based microscopes the use of a single frequency. Thereby, in very short measuring times an overview image is obtained to select the region of interest, which is than in a second step detailed analyzed. This will reduce the time for tissue classification down to very few minutes which is within clinical timeframes.

An additional approach to reduce the experimental workload can be achieved using only few discrete marker-frequencies, as previously reported for the first generation Spero instrument^20^. In this approach, few marker wavenumbers were selected and used for classification of the normal and cancerous epithelium. In our study, this strategy is still hampered, due to the required Mie correction. New pre-processing algorithms have to be developed to apply this promising approach.

Since the QCL based microscope shows several advantages over the previous FTIR based instruments, QCLs based imaging are a promising alternative in biomedical imaging applications. However, coherence effects on sample edges and the sample itself must be addressed in future studies to obtain not only tissue classification but also comparable spectral quality to FTIR. The studies performed by the Bhargava group addressed this point very carefully, elaborated nicely the coherence and proposed coherence handling for better comparability to FTIR^23–25^. Furthermore, FTIR imaging could also be accelerated by decreasing the number of scans and reducing the noise during pre-processing. We will further demonstrate the clinical applicability of our approach using in the next step a much larger patient cohort in a multi-centered study. Additionally, future studies should focus on different pathologies, and should demonstrate the clinical relevance of the analysis for broader applications. The results of our study may allow the translation of the IR based imaging into clinics, since we demonstrated that the time required for spectral imaging decreased to the level of that required for other standard procedures, such as H&E or IHC, but can be automated and is operator/observer independent.

The appropriate classification procedure, computing time, and usability represent the key issues that need to be solved for clinically application, but the reliability of the system should be considered as well. The diagnostic system must be able to analyze the samples independently from the original training setup, the observer, and the operator. These include the dependency on the workflows in different clinics and the use of different instruments. The effects of using different FTIR imaging instruments and operating personnel were previously investigated^29^, but a similar study has not been performed for the QCL based instruments. Therefore, in this study we demonstrated the robustness of our approach by confirming the obtained results using another Spero QT microscope, which has been used for the validation only. Furthermore, all measurements were performed independently by three operators, to examine the potential issues in clinical application and determine whether the automated annotation approach is transferable to other Spero QT microscopes using the same classifier. Sensitivity and specificity are slightly altered by this. This demonstrates the transferability of our classifier between different instruments and remarks an important prerequisite for translation of IR based tissue annotation to the clinic.

To summarize, we addressed in our study the major issues to overcome translation of infrared based label-free imaging to the clinics. We successfully reduced the measuring time fitting the pathological workflow with lowest possible intra- and inter-operator/observer variability. Further approaches to reduce the measuring time even more and the computational workload are also presented. In order to increase acceptance in the medical/pathological community in the next step larger studies of unmet clinical needs will be addressed. This may propel infrared based label-free and automated tissue classification into clinical routines. Furthermore, by IR imaging guided laser capture microdissection the approach can be used for largely improved biomarker search^14^. IR imaging is a non-destructive, label-free, and now rapid technique for tissue classification and biomarker research.

## Methods

### Ethical statement

All methods and experimental protocols were approved by the relevant institutional review boards (registration number 4453-12, Ethics Commission, Faculty of Medicine, Ruhr-University Bochum). Furthermore, all enrolled patients provided their informed consent. All procedures were conducted in accordance with the approved guidelines and regulations for human experimental research.

### Sample preparation

We obtained 120 samples of 110 patients from the Institute of Pathology, Ruhr University Bochum, Germany, including 20 tumor-free tissue sections and 100 tumor samples. The samples were randomly chosen from a cohort including patients with primary tumor UICC-Stage II and III and older than 18 years. Tumor-free samples were obtained from 10 patients, both tumor-free and tumor samples were obtained from 10 patients, while tumor samples were obtained from 90 patients. The samples were collected during surgery and, following the fixation, they were handled according to the standardized protocols used at the Institute of Pathology. Colorectal cancer formalin-fixed paraffin-embedded (FFPE) tissue blocks were cut into 7-µm thick sections and floated onto Leica frame slides with a 1.4-µm thick PET membrane. Tissue was dewaxed using established standards in groups of 10 before spectral data acquisition.

### Spectral data acquisition

All measurements were performed on two Spero QT (Daylight Solutions, CA, USA) QCL based microscopes. We installed an additional purge air diffuser to the sample chamber of the Spero QT to reduce equilibration time and modified the microscope stage to obtain enough space to analyze two slides in a row. Data acquisition was performed using the chemical vision software (Daylight Solutions, CA, USA). We used the installed 4 × 0.3 NA objective for large scale measurements covering a 2 × 2 mm^2^ FOV. The mounted uncooled microbolometer focal plane array (FPA) detector is 480 × 480 pixels large, resulting in the pixel size of 4.25 × 4.25 µm. Full spectra were obtained in the range of 1800–948 cm^−1^ with a spectral resolution of 2 cm^−1^ in the transmission mode. These 120 clinical samples were analyzed in less than 100 h.

### Spectral processing and analysis

The spectral maps were pre-processed using the previously described workflow^11^. Strong spectral artifacts that may emerge from the folds or cracks in the tissue were eliminated by quality control based on the signal-to-noise ratio and the integral of the amid I band. The remaining spectra were subjected to a Mie correction based on EMSC in the wavenumber range of 1800 to 950 cm^−1 ^^30,31^. Tests showed that one iteration step does not affect the results in our case. Unsupervised clustering (*e.g*., hierarchical, HCA, or k-means clustering) was performed on smoothed (9-point Savitzky-Golay) second derivative spectra. The supervised RF classification was based on unsmoothed Mie corrected spectra as previously described^11^. For both methods the analysis was performed on the fingerprint region from 1750 to 1000 cm^−1^ due to lower laser energy on the edges of the QCLs.

### Classifier set-up and spectral database generation

In this study we used the workflow established and described in our previous publications^11–13^. RF classifier was shown to be a robust and reliable classifier for biomedical imaging^12,32–34^, and we used two consecutive RF analysis. During the training stage, a spectral database was generated, and the pathological regions, infiltrating inflammatory cells, muscles, lumen, crypts, and connective tissue were identified in the representative spectra. The second database contained the spectral signatures for cancerous, inflammatory, and necrotic areas and inflammatory cells identified by the first RF. It was not possible to describe the muscle tissue spectrally in one class, and we defined several muscle classes in the spectral databank. Spectra classified as pathological were subjected to the second RF analysis. Thirteen patient samples were used for training, and 97 patients for the validation. Both RFs were built with 500 trees using 16 spectral features randomly chosen per decision in the trees. After obtaining the lower signal-to-noise ratio and baseline effects, the spectral hypercube was reduced to 1760-998 cm^−1^ range, with 2 cm^−1^ spacing for both levels of RF classification. Thus, 382 wavenumbers were used for RF classification. The Gini variable importance for both RF classifiers were shown in Fig. S10. The most significant features for both RFs are in the Amid I, Amid II, and between 1300 to 1000 cm^−1^. These results correspond to the previous results with FTIR imaging from us and other groups. All computations were performed in MATLAB (Mathworks, USA). The final tissue annotation was provided as index color images and compared with that of the corresponding H&E-stained tissue images. The pathologists at the Institute of Pathology, Ruhr University Bochum, supplied their section analyses as well.

## Electronic supplementary material

Supplementary material
