# Supplementary material for: Quantum Cascade Laser-Based Infrared Microscopy for Label-Free and Automated Cancer Classification in Tissue Sections
Source: Sci Rep. 2018 May 16;8:7717. doi: 10.1038/s41598-018-26098-w (PMC5955970; doi:10.1038/s41598-018-26098-w)
Supplement: Supplementary file 1 — Supplementary material [file 41598_2018_26098_MOESM1_ESM.docx]

**Quantum Cascade Laser-Based Infrared Microscopy for Label-Free and Automated Cancer Identification in Tissue Sections**

**Authors:** Claus Kuepper^1^†, Angela Kallenbach-Thieltges^1^†, Hendrik Juette^2^, Andrea Tannapfel^2^, Frederik Großerueschkamp^1^ and Klaus Gerwert^1^*

**Affiliations:**

*^1^* *Chair of Biophysics, Faculty of Biology and Biotechnology, Ruhr University Bochum, Germany.*

*^2^ Institute of Pathology, Ruhr University Bochum, Germany.*

* To whom correspondence should be addressed: gerwert@bph.rub.de

† These authors contributed equally.

**List of Supplementary Materials**

Fig. S1. The surface plots represent the 100% line at 1500 cm-1 for one 480 x 480 pixels detector field.

Fig. S2 comparison of QCL and FTIR imaging index color images from the classifiers and corresponding H&E stained image.

Fig. S3 Comparison of spectra between FTIR and QCL based IR imaging.

Fig. S4 Mean spectra of the training data for selected classes with standard deviation.

Fig. S5 Mean spectra of the training data for selected classes with difference.

Fig. S6 Structure of RF classifiers utilized for the annotation of colonic tissue sections.

Fig. S7 Detailed representation of the infiltrating inflammatory cells and lymph follicles.

Fig. S8 Spectral representation of infiltrating inflammatory cells and lymph follicles for FTIR and QCL based RF classifier with standard deviation

Fig. S9 QCL-based images showing the applicability of the RF classification system following the reduction of spectral resolution.

Fig. S10 Gini importance of the 1^st^ and 2^nd^ RF

**Supplementary Materials:**

**
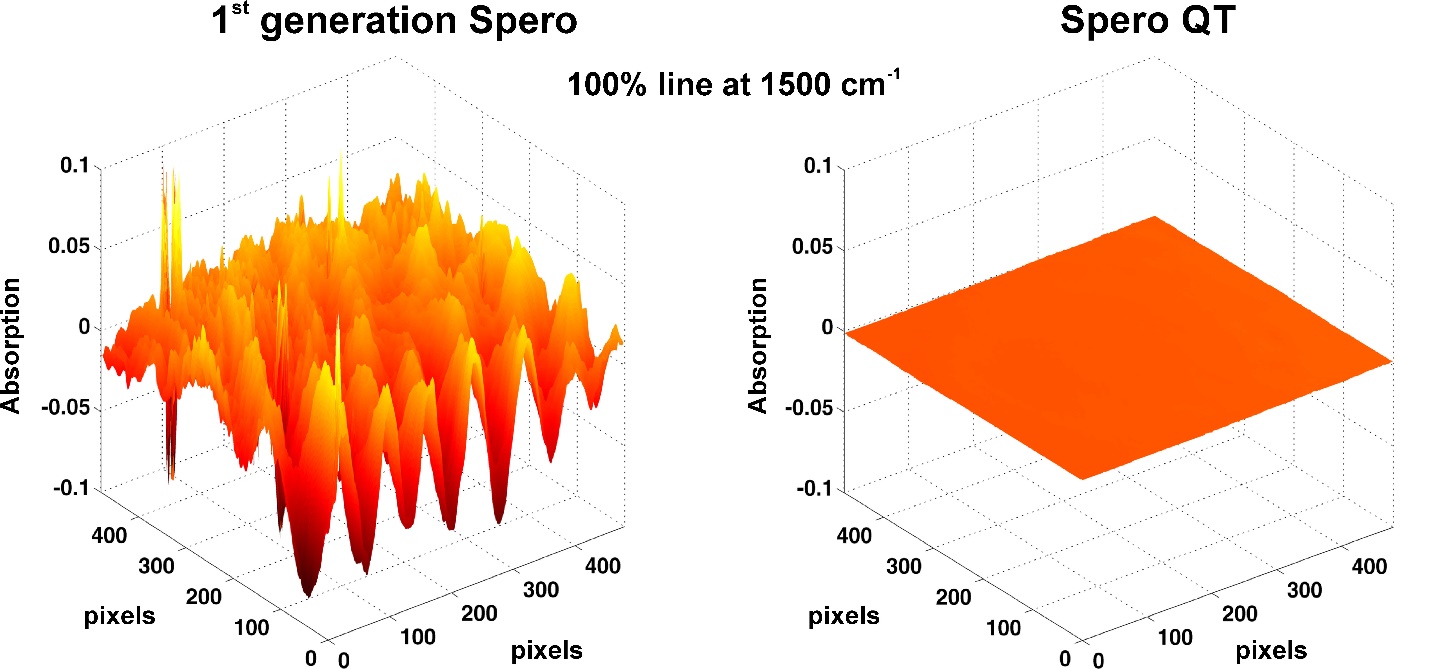
**

Fig. S1. The surface plots represent the 100% line at 1500 cm^-1^ for one 480 × 480 pixels detector field. Left, the first-generation Spero result (noise level, 10^-1^). Right, Spero QT result (noise level, 10^-3^). The entire system has been optimized, including QCL stability, allowing the lower variance of fringes caused by the microscope optics (right, observed as the sinus-shaped pattern). Lowering the variances caused by the instrument is an important step towards clinical diagnostics.

**
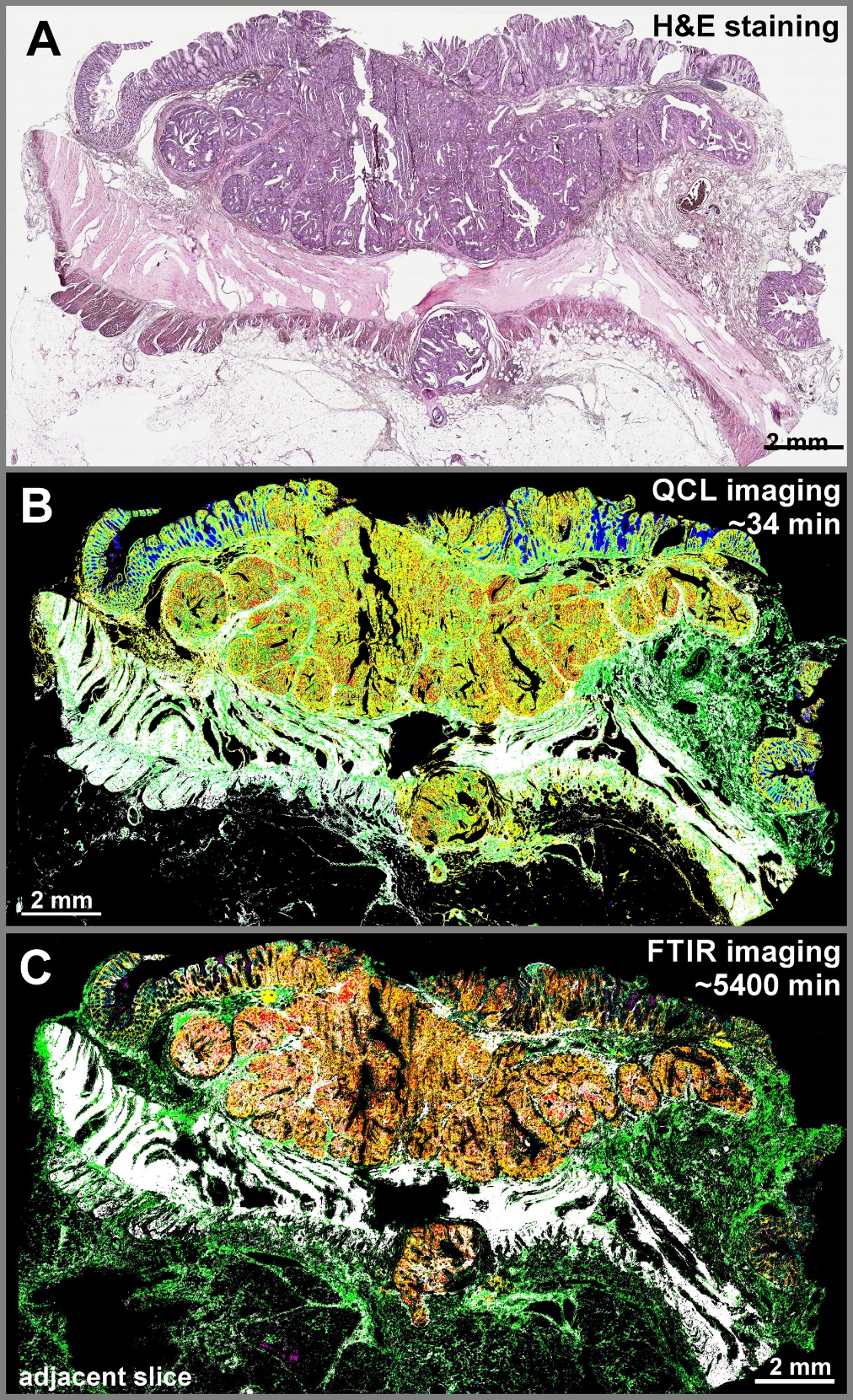
**

Fig. S2. Comparison of the infrared based tissue analysis. Colorectal cancer tissue analysis using H&E staining as Gold standard (A), Spero QT system (B), and FTIR-based imaging system (C). The listed times illustrate the duration of the measurements. Red, pathological region comprising of tumorous regions and infiltrating inflammatory cells; white, muscles; green, connective tissue; cyan, crypts and blue, lumen. The comparison of the images demonstrates convincingly, that the QCL IR imaging results are in nice agreement with those obtained using the FTIR imaging. The observed deviations seem to be caused mainly by the use of an adjacent slice and the training of the FTIR classifier on samples of another study which could slightly differ in sample handling and processing. Furthermore, the previously FTIR classifier is performing the classification in one step which is less accurate by means of tumor detection. While the improved classifier for the QCL imaging recognizes infiltrating inflammatory cells in the first level random forest (RF) and cancerous regions in the second level RF which allows a much more accurate classification.


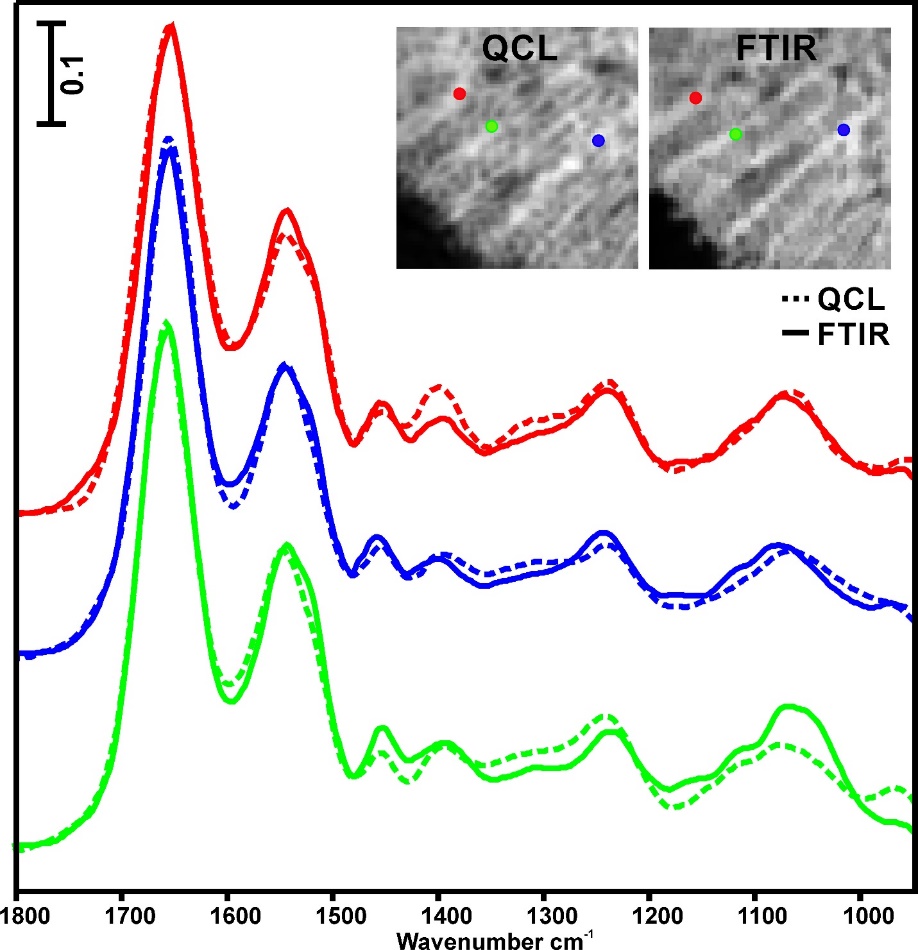


Fig. S3. Comparison of spectra between FTIR and QCL based IR imaging. Three points were selected in an FTIR and QCL based imaging measurement of the same sample. The corresponding spectra are shown. Slight differences are visible that occur due to different pixel size, accuracy of the overlay, or coherence effects. The differences have to be addressed in future pre-processing optimization to make the instruments more comparable to FTIR in spectral quality. Using the instruments for tissue classification is still possible.

**
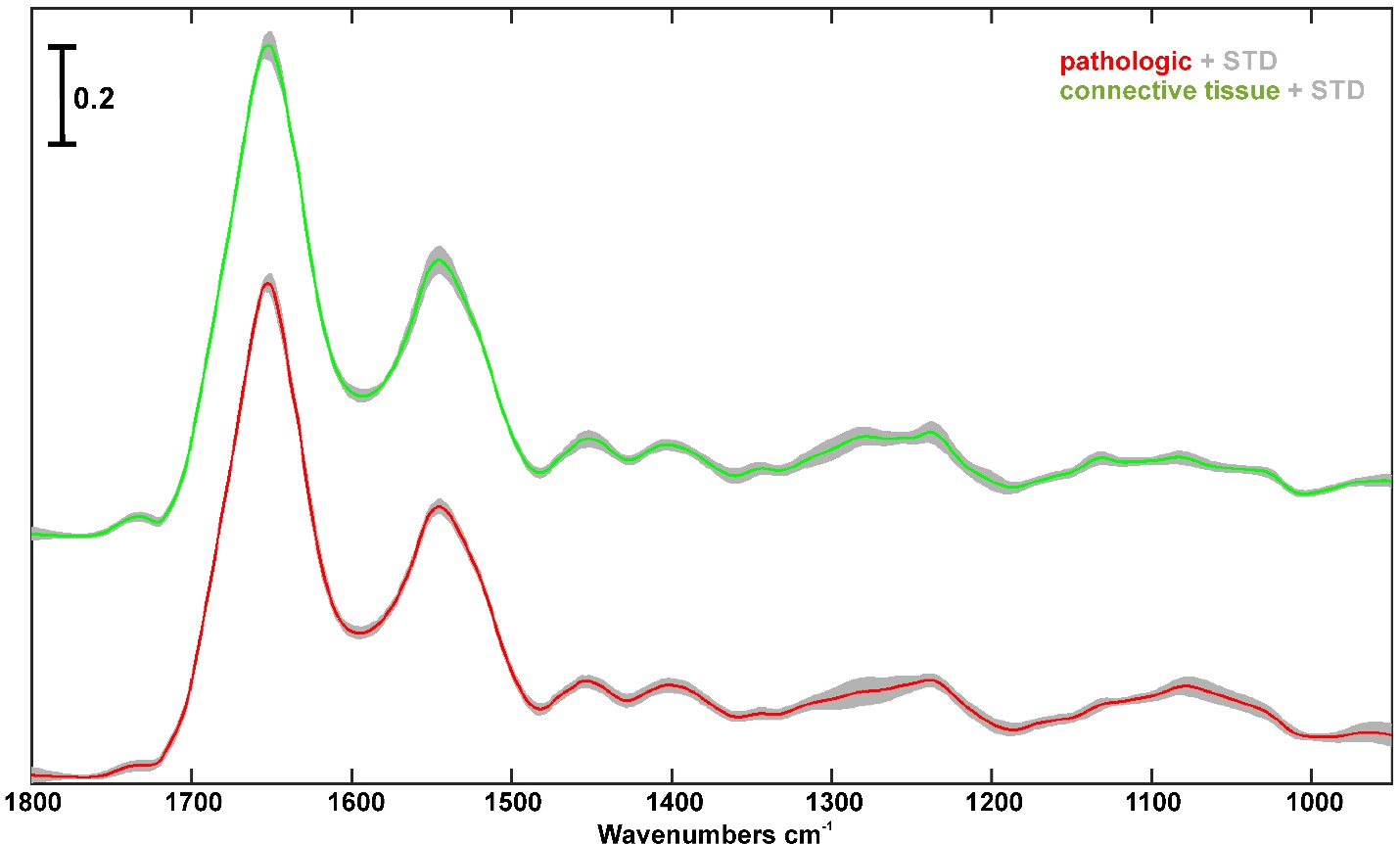
**

Fig. S4. Mean spectra of the training data for selected classes with standard deviation. Red spectrum, the mean of all pathological region spectra. Green spectrum, the mean of connective tissue training spectra. The grey area marks the standard deviation over the training data. The highest STD is obtained from 1300 to 1250 cm^-1^. This could be correlated to coherence effects on the sample. Since the spectral variance is represented in the training data tissue classification is still possible.


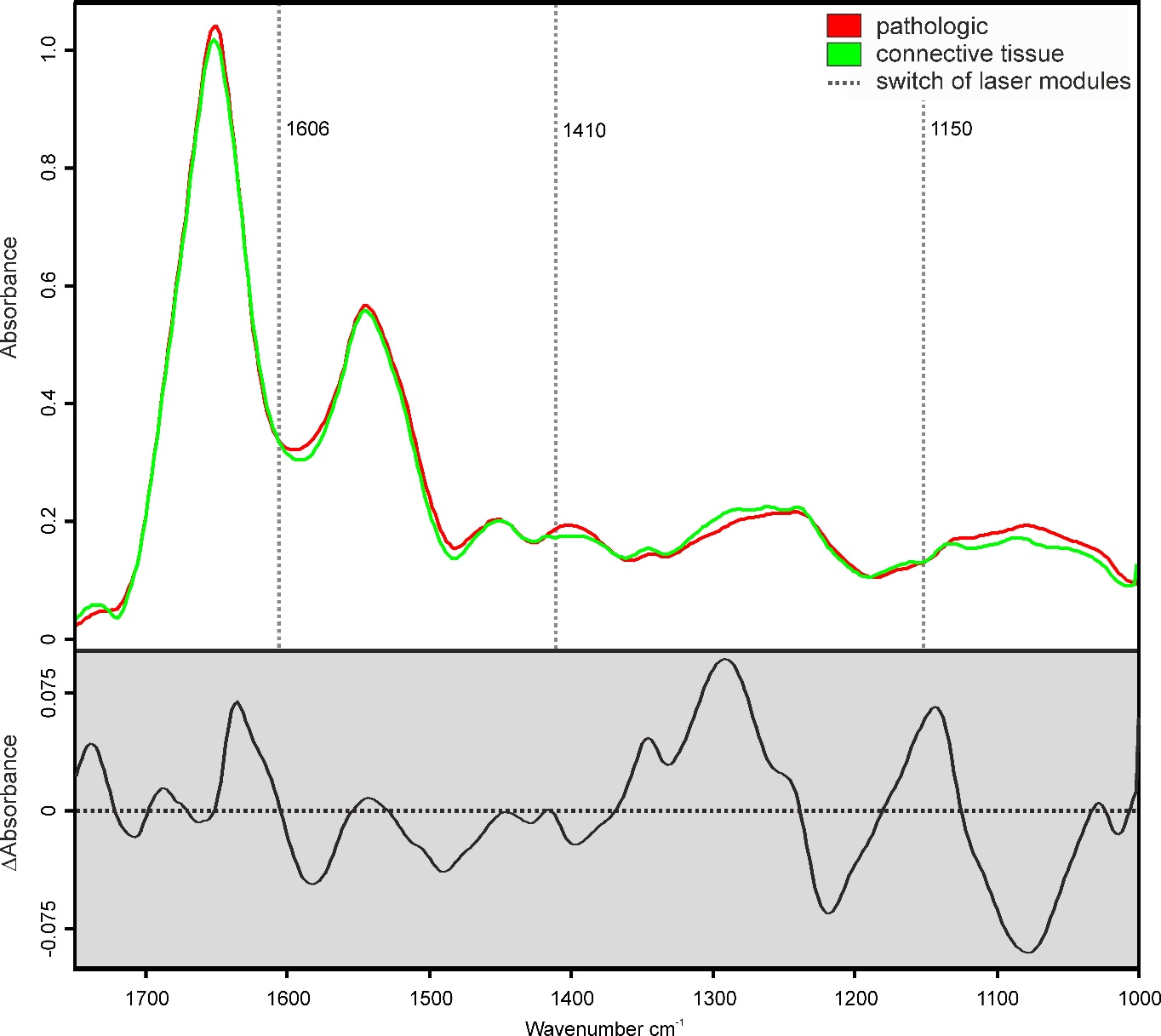


Fig. S5. Mean spectra of the training data for selected classes with difference. Red spectrum, the mean of all pathological region spectra. Green spectrum, the mean of connective tissue training spectra. The dotted lines, module changes during QCL tuning. The Spero QT has four laser modules covering the fingerprint region. The first supports the frequencies from 1150 to 920 cm^-1^, the following emits frequencies at 1410 to 1150 cm^-1^, the third module covers the range of 1606 to 1410 cm^-1^, while the last module completes the spectrum, covering the frequencies from 1800 to 1606 cm^-1^. Black line, the difference between the pathological and connective tissue samples.


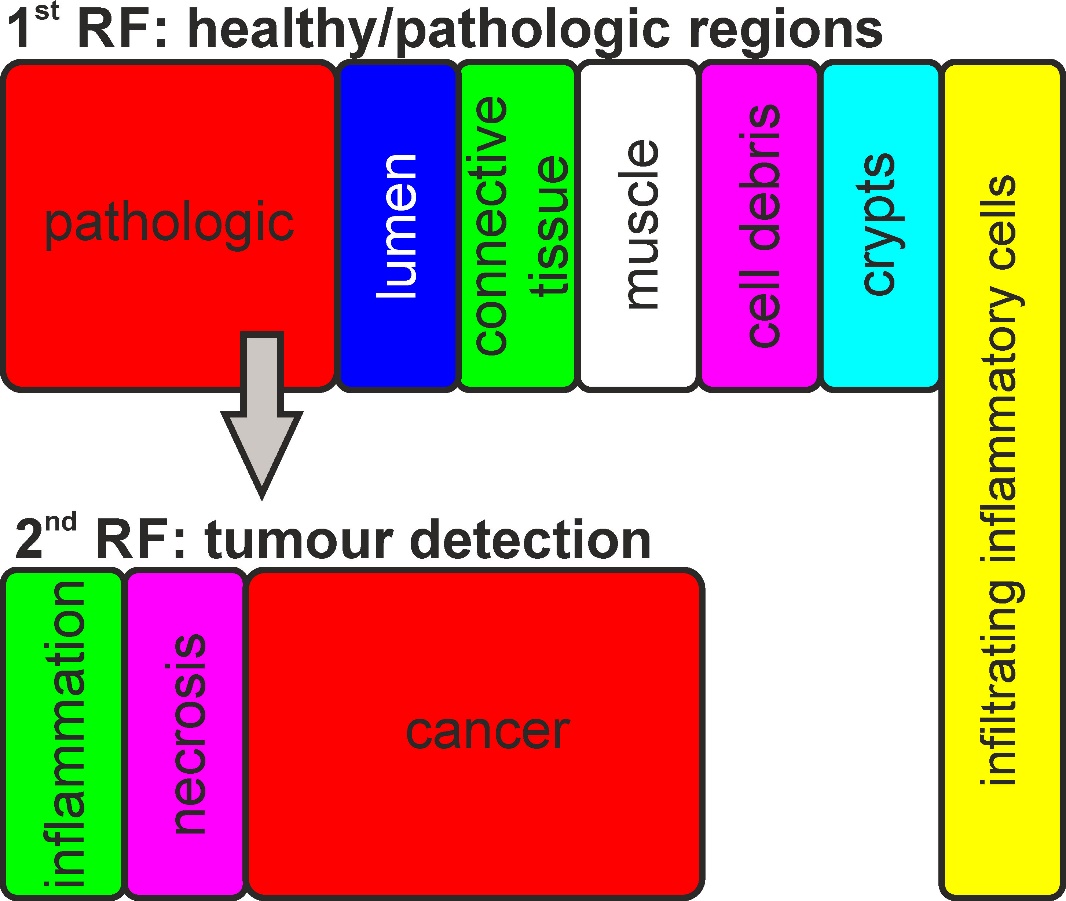


Fig. S6. Structure of random forest (RF) classifiers utilized for the annotation of colonic tissue sections. The first layer identifies pathological tissue and healthy areas, including the connective tissue (green), cell debris (magenta), crypts (cyan), lumen (blue), muscular structures (white), and infiltrating inflammatory cells (yellow). For the better morphological representation, infiltrating inflammatory cells are presented in the index color image of the second RF as well. Spectra identified as pathological (red) at the first layer are subsequently analyzed at the second RF, and labeled as cancerous areas (red), necrotic (magenta), and inflamed (green) regions.


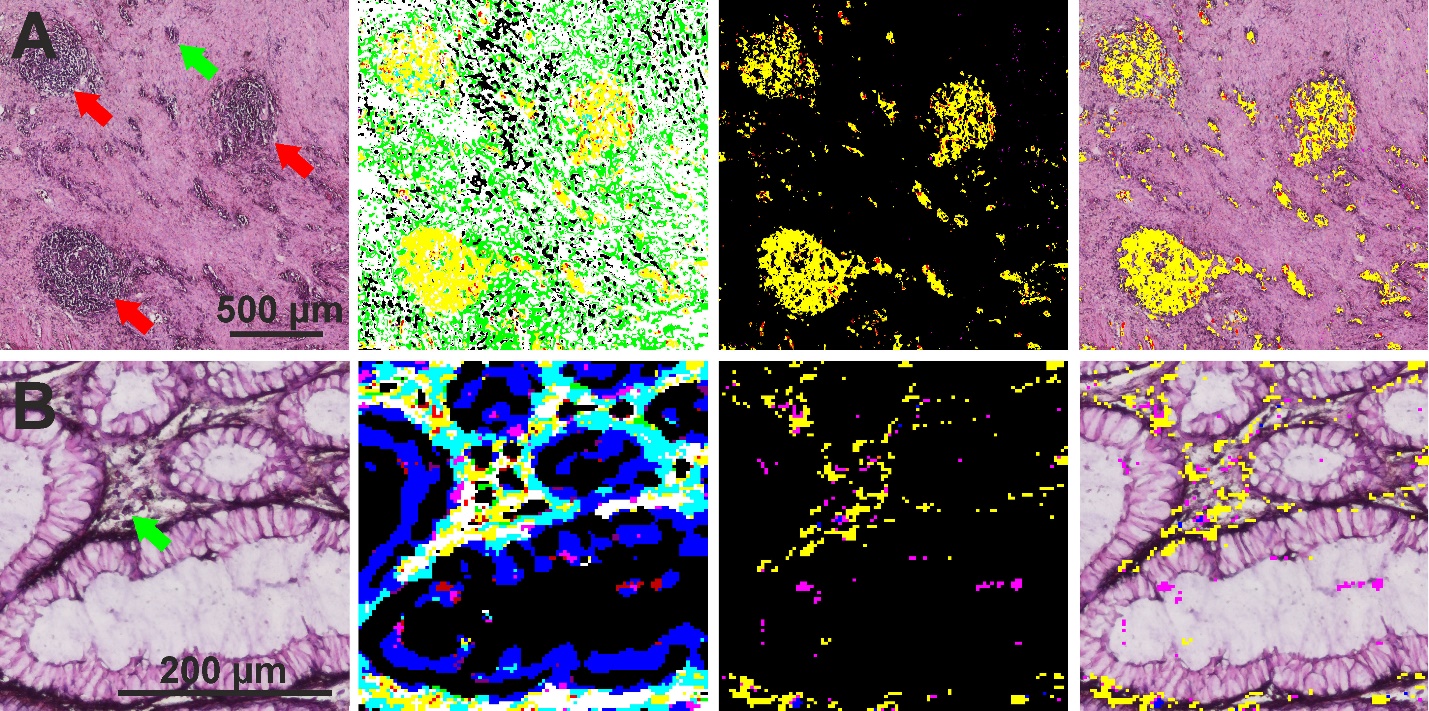


Fig. S7. Detailed representation of the infiltrating inflammatory cells and lymph follicles. A, three lymph follicles (red arrow) and infiltrating inflammatory cells are represented in this tissue stain. The RF classifier identifies these regions with high accuracy. The first index color image represents the first RF with connective tissue in green, muscle in white and inflammatory cells/lymph follicle in yellow. The second index color image shows the yellow class solely and the third the overlay with the HE stain. The high accuracy in classification is well recognizable. B, shown are crypts with infiltrating inflammatory cells (e.g. green arrow). The first index color image represents the first RF with crypts in blue, infiltrating inflammatory cells in yellow, muscle in white, and pathologic regions in red. No tumor was detected in the second RF for the pathologic region (magenta = necrosis). The overlay with the HE stained image underlines the accurate tissue identification.


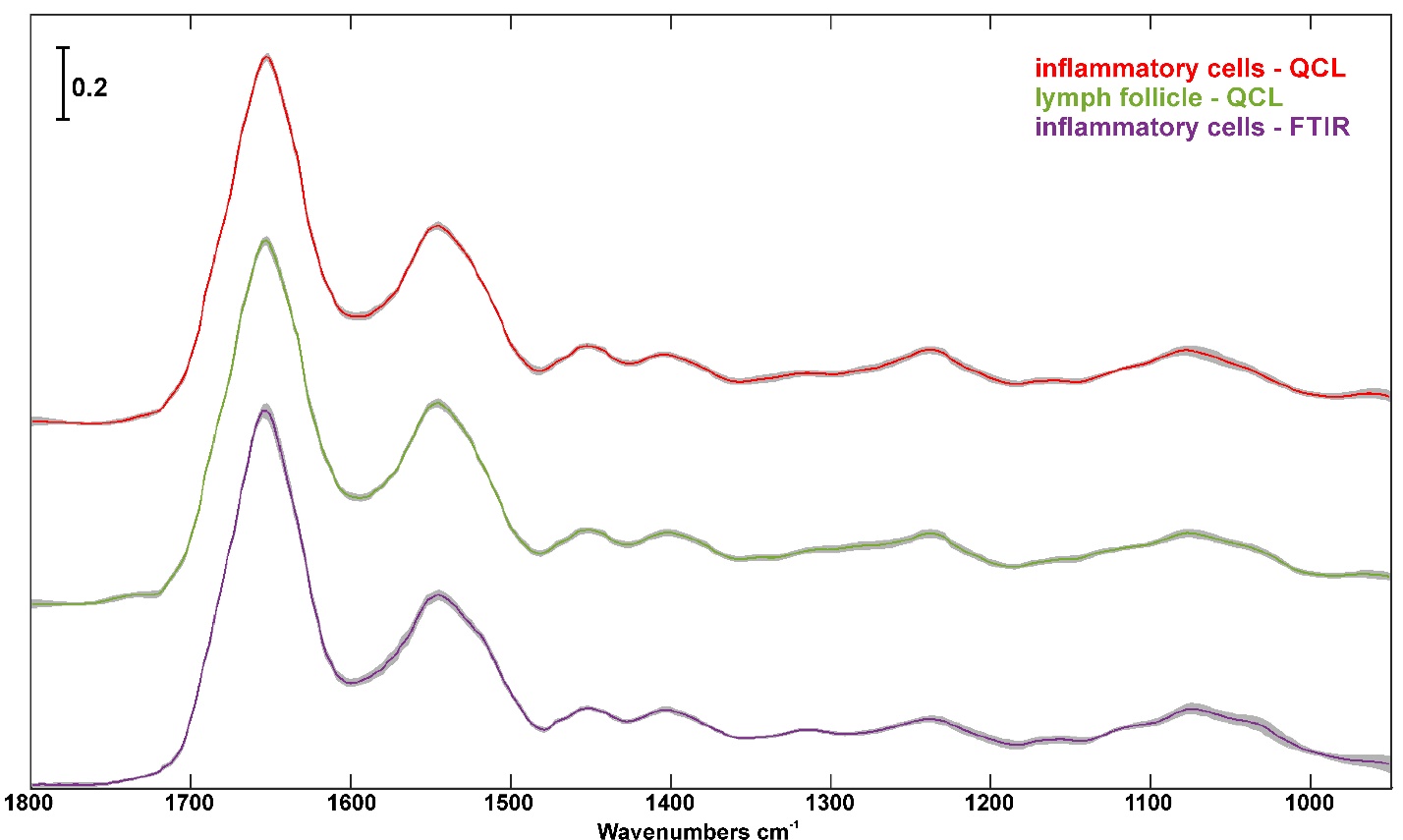


Fig. S8. Spectral representation of infiltrating inflammatory cells and lymph follicles for FTIR and QCL based RF classifier with standard deviation. Shown are the mean spectra for spectral classes that are occurring for infiltrating inflammatory cells and lymph follicles. The mean of the infiltrating inflammatory cells class is shown for FTIR in purple and for QCL in red. Furthermore, for QCL a spectral class adjusted for lymph follicles is shown. The grey area represents the standard deviation of each spectral class. The spectral classes are quite similar between FTIR and QCL. This proofs the usability of QCL based imaging for tissue classification.


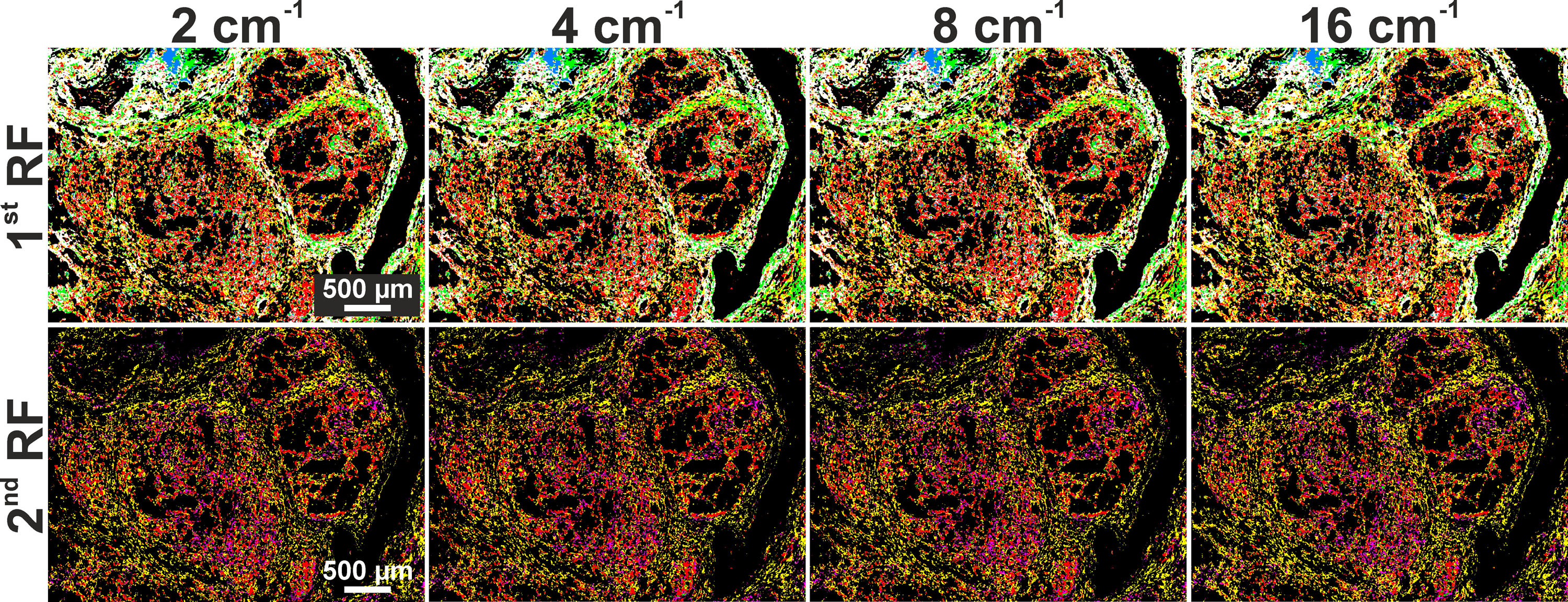


Fig. S9. QCL-based images showing the applicability of the RF classification system following the reduction of spectral resolution. Far left, standard settings at 2 cm^-1^; top lane: red, pathological regions; yellow, the invading inflammatory cells; white, the surrounding muscles; green, connective tissue. All regions marked red using the first level RF were further analyzed by the second level RF. Bottom lane: red, tumor cells; magenta, necrotic regions; yellow, infiltrating inflammatory cells. The decrease in the spectral resolution did not decrease the quality of the results.


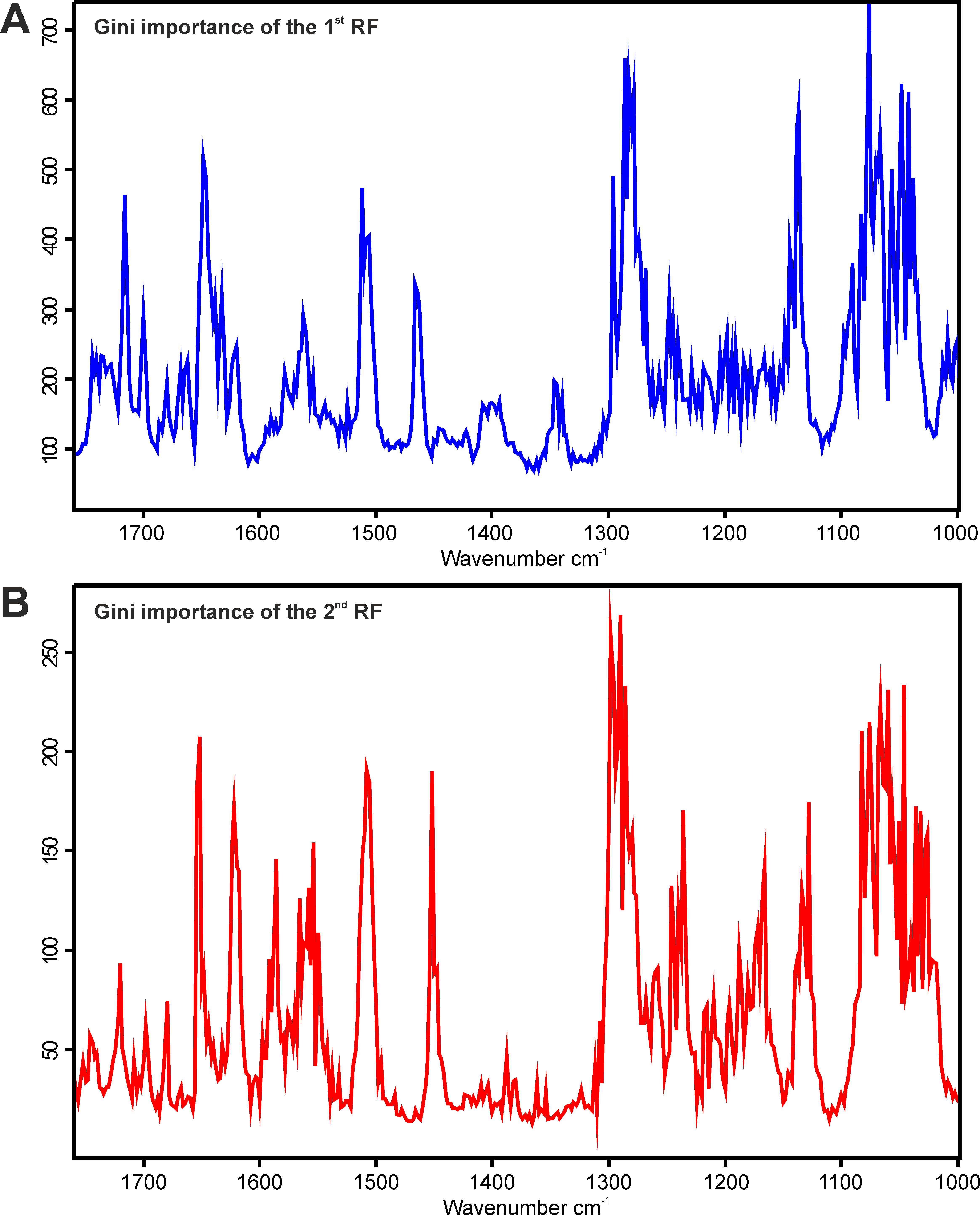


**Fig. S10. Gini importance of the 1st and 2nd RF.** Shown are the Gini variable importance obtained from the random forest classifiers developed for automated colorectal cancer classification. The most significant features for both RFs are in the Amid I, Amid II, and between 1300 to 1000 cm^-1^. These results correspond to the previous results with FTIR imaging from us and other groups. The random forest algorithm of Leo Breiman, used here, selects randomly 16 features for each decision node. Therefore, no user dependent feature selection is needed. This is a benefit of RF algorithm and results in their robustness against over fitting and unbalanced class sizes.
